# Post COVID-19 condition after delta infection and omicron reinfection in children and adolescents

**DOI:** 10.1016/j.ebiom.2023.104599

**Published:** 2023-05-05

**Authors:** Nina Urke Ertesvåg, Arild Iversen, Bjørn Blomberg, Türküler Özgümüş, Pramila Rijal, Elisabeth Berg Fjelltveit, Rebecca Jane Cox, Nina Langeland, Bergen COVID-19 research group, Kjell Haug, Helene Sandnes, Kristin G-I Mohn, Jan Stefan Olofsson, Marianne Sævik, Christopher James Brokstad, Kanika Kuwelker, Kristin Heienberg

**Affiliations:** a Influenza Centre, University of Bergen, Bergen, Norway; b Chief Municipal Doctor's Office, Bergen Municipality, Bergen, Norway; c Department of Clinical Science, University of Bergen, Bergen, Norway; d Department of Medicine, Haukeland University Hospital, Bergen, Norway; e National Advisory Unit for Tropical Infectious Diseases, Haukeland University Hospital, Bergen, Norway; f Department of Global Public Health and Primary Care, University of Bergen, Bergen, Norway; g Center for Translational Immunology, Chinese Academy of Medical Sciences Oxford Institute, Nuffield Department of Medicine, University of Oxford, Oxford, UK; h MRC Human Immunology Unit, MRC Weatherall Institute, John Radcliffe Hospital, Oxford, UK; i Department of Microbiology, Haukeland University Hospital, Bergen, Norway

**Keywords:** Post COVID-19 condition, SARS-CoV-2 infection, Antibody, Delta variant, Omicron variant, Children and adolescents

## Abstract

**Background:**

The burden of COVID-19 in children and adolescents has increased during the delta and omicron waves, necessitating studies of long-term symptoms such as fatigue, dyspnoea and cognitive problems. Furthermore, immune responses in relation to persisting symptoms in younger people have not been well characterised. In this cohort study, we investigated the role of antibodies, vaccination and omicron reinfection upon persisting and long-term symptoms up to 8 months post-delta infection.

**Methods:**

SARS-CoV-2 RT-PCR positive participants (n = 276, aged 10–20 years) were prospectively recruited in August 2021. We recorded the major symptoms of post COVID-19 condition and collected serum samples 3- and 8-months post delta infection. Binding antibodies were measured by spike IgG ELISA, and surrogate neutralising antibodies against Wuhan and delta variants by the hemagglutination test (HAT).

**Findings:**

After delta infection, persisting symptoms at 3 months were significantly associated with higher delta antibody titres (OR 2.97, 95% CI 1.57–6.04, p = 0.001). Asymptomatic acute infection compared to symptomatic infection lowered the risk of persisting (OR 0.13, 95% CI 0.02–0.55, p = 0.013) and long-term (OR 0.28 95% CI 0.11–0.66, p = 0.005) symptoms at 3 and 8 months, respectively. Adolescents (16–20 years) were more likely to have long-term symptoms compared to children (10–15 years) (OR 2.44, 95% CI 1.37–4.41, p = 0.003).

**Interpretation:**

This clinical and serological study compares long-term symptoms after delta infection between children and adolescents. The association between high antibody titres and persisting symptoms suggest the involvement of an immune mechanism. Similarly to adults, the dominant long-term symptoms in children are fatigue, dyspnoea and cognitive problems.

**Funding:**

This work was funded by the 10.13039/501100003506Ministry of Health and Care Services, Norway, the University of Bergen, Norway and 10.13039/501100003506Helse Vest, Norway (F-12621).

**Research in context**

**Evidence before this study**

We searched PubMed, MEDLINE and preprint repositories from May 9th, 2022, to January 5th, 2023, for publications on long-term symptoms after COVID-19 in children and adolescents without any language restrictions. Search terms were “COVID-19”or “SARS-CoV-2” adjacent to long COVID or post COVID-19 condition synonyms, combined with various search terms for children and adolescent and age filters (10–20 years). We further extended this search to include key words for available COVID mRNA vaccines, comprising preprint repositories. Finally, we searched for “immun∗” or “serolog∗ or antibod∗ after COVID infection or vaccination in the relevant age group. We also found relevant references via citation searching. The major paediatric long-COVID studies are large, cross-sectional epidemiological studies that investigate persisting symptoms after SARS-CoV-2 infection with the ancestral or alpha strain, using online apps for data collection. However, prospective systematic studies including immune responses are largely lacking. The general conclusion drawn from current research is that long-term symptoms in young children are rare, but more common in adolescents. Other risk factors are reported to be female sex and acute SARS-CoV-2 symptoms. We could not find any published articles on how vaccination impacted symptoms in a young cohort.

**Added value of this study**

In our observational paediatric study, we have characterised risk factors for long-term symptoms, using the WHO post COVID-19 condition definition, after delta infection and omicron reinfection. The study was designed to compare symptoms between children and adolescents. We collected blood samples from a subgroup, providing a unique opportunity to connect persisting symptoms and reinfections with immunological data. We found that acute symptomatic infection and higher antibody titres correlated with post COVID-19 condition. Adolescents were more likely to report long-term symptoms than children, including fatigue (44% vs 19%), dyspnoea (25% vs 16%) and cognitive symptoms (36% vs 16%), which often persisted over time. Omicron infection resulted in increased respiratory and systemic symptoms, particularly in children.

**Implications of all the available evidence**

While mortality and severe acute illness is uncommon in children and adolescents, persisting symptoms may be a major risk in these young people. The societal impact may be further escalated by emergence of new variants with increased infectivity, as observed by high rates of omicron reinfection in our study. To mitigate long-term COVID sequela, more studies are needed on pathophysiological mechanisms in vulnerable groups, including children and adolescents.

## Introduction

Infection with severe acute respiratory syndrome coronavirus 2 (SARS-CoV-2) can cause long-term symptoms, commonly known as post COVID-19 condition or long COVID.^1^ This condition has predominantly been characterised in adults and fewer studies have focused on children and adolescents.^2^^,^^3^ Consequently, no paediatric long COVID definition has been determined, but a recent initiative suggested the following definition: at least one persisting symptom with a minimum 3-month duration, which impacts everyday functions.^4^ Our evolving understanding is that risk factors for paediatric long-term symptoms are acute symptoms, post-puberty age and female sex.^5^

Knowledge about long-term symptoms comprises mostly the ancestral Wuhan-Hu-1 (Wuhan) and alpha strain, while data on long-term symptoms after delta and omicron infection is limited.^6^ Significantly higher upper respiratory tract viral loads were associated with the delta variant.^7^ Previous studies with earlier variants have found low prevalence of long-term symptoms in children <16 years compared to adults^8^^,^^9^ or uninfected controls.^10^, ^11^ Younger people have experienced the burden of new infections with delta and omicron, and therefore the risk of post-COVID complications may have increased in this age group.^12^ Although COVID vaccines were licensed, adolescents were often not vaccinated before the delta wave due to prioritised vaccine roll-out, partially explaining high infection rates in this population.^13^ It is unknown if COVID vaccination decreases persisting symptoms in younger age groups, but consensus of a protective vaccine effect in adults is growing.^14^

Previous studies on post COVID-19 condition have found an association between the SARS-CoV-2 spike IgG antibodies and persisting symptoms in adults,^15^ even after mild disease.^8^^,^^9^ We have previously shown that a rapid, low-cost receptor-binding domain (RBD)-specific hemagglutination test (HAT) is highly correlated with neutralising antibodies, including antibodies to delta, and may be used as a correlate for neutralising antibodies.^16^

We hypothesised that the higher viral load reported after delta infections would impact persisting symptoms in children and adolescents. We investigated if an association between immune responses and long-term symptoms could be found in children, similarly to adults. In this study, we addressed these knowledge gaps by following a prospectively recruited cohort of children after mild SARS-CoV-2 delta infection.

## Methods

### Study design

During the first delta wave in Bergen municipality, children and adolescents (n = 276, 40% of eligible participants) aged 10–20 years who had SARS-CoV-2 infection confirmed by RT-PCR in a nasopharyngeal or oral swab were recruited, from August 1st to September 16th, 2021 (Supplementary Table S1). Local recommendations for testing were symptoms of acute respiratory infection or close contact with a confirmed SARS-CoV-2 positive person. Questionnaires were answered at recruitment (baseline), 3 and 8 months after initial delta infection (Supplementary Fig. S1). Serum samples were collected at 3- and 8-months post infection from n = 88 and n = 87 participants, respectively.

### Ethical considerations

The study was approved by the Regional Ethics Committee of Western Norway (#118664) and registered in ClinicalTrials.gov (NCT04706390). All individuals provided written or digital informed consent. For children aged 15 years or younger, parents or legal guardians signed the informed consent.

### Clinical data collection

Baseline data were collected by telephone interviews of children or their parents (children <16 years) and stored in electronic case report forms (eCRFs) in the Research Electronic Data Capture database (REDCap®), Vanderbilt University, Nashville, Tennessee. The first follow-up at 3 months post-infection (median 104 days, interquartile range (IQR) 62–109 days) involved a subgroup of participants (n = 89) who were willing to come to the study outpatient clinic and donate a blood sample (n = 88). The second follow-up at 8 months post-infection (n = 204, median 245 days, IQR 205–254 days), was a combination of online responses, telephone interviews and in-person at our hospital's outpatient clinic. All participants were invited to answer a follow-up online questionnaire. A link to the eCRF was sent by SMS to participants ≥16 years or to their parents if less than 16 years. If they were unable to attend the clinic, participants (51%) were interviewed by telephone with the same questionnaire.

Questionnaires at baseline, 3 and 8 months recorded demographic information, comorbidities, medication, COVID-19 vaccination, any reinfection, and up to 18 COVID-19 related symptoms (see Supplementary Table S2). The WHO-definition of post COVID-10 syndrome,^1^ where fatigue, dyspnoea and cognitive symptoms are highlighted, were utilised when defining persisting (3 month) and long-term (8 month) symptoms. Alternative diagnoses were excluded by collecting information on comorbidities, and specifically asking if the children had been diagnosed with any new medical condition during the last 8 months at the 8-month follow-up. If a participant reported any persisting or long-term symptoms, not explained by any other diagnosis, the participant was considered to fulfil the criteria for post COVID-19 condition. All participants answered dichotomized yes/no questionnaires with questions about persisting COVID-19 related symptoms at follow-up visits. At 3 months, the validated 11-questionnaire Chalder Fatigue Scale (CFS)^9^ with graded responses was used to assess physical and mental fatigue in participants aged ≥16 years, while a modified shortened version of CFS was answered by all participants at the 8-month follow-up.

Up to February 2022 a positive SARS-CoV-2 antigen test result was confirmed by RT-PCR, but later the national health authorities accepted rapid antigen tests as a verified case of COVID-19, and this is reported as a positive test in our cohort. From December 30th, 2021, all recorded reinfections were defined as omicron infections, based on surveillance data from the Norwegian Institute of Public Health. Participants were not screened for omicron reinfections, but actively asked if they had had SARS-CoV-2 infection. During this period, active testing was conducted by the municipality with free lateral flow tests provided to all Norwegians and positive tests confirmed by centralised SARS-CoV-2 PCR testing. During recruitment, the national health authorities recommended COVID-19 vaccination for all age groups 16 years and older. Subjects vaccinated <14 (n = 7) and <3 (n = 4) days pre-infection were considered unvaccinated when analysing the impact on symptoms, and antibody responses, respectively. The two different cut-offs were based on immunological responses. Vaccination after a priming stimulus (or infection followed by vaccination) may recall memory B cells within 3–5 days. The cut-off was therefore set at three days. However, when it comes to symptoms, we chose a minimum of 14 days to give sufficient time for the antibody responses to reach a plateau, before measuring its potential effect on symptom outcomes.

Source data are available upon reasonable request to the corresponding author.

### Serum samples

Serum samples were collected at follow-up visits at 3 and 8 months (Supplementary Fig. S1). At the first follow-up, 88 participants provided serum samples, and 87 did so at the second visit. Clotted blood was centrifuged and sera separated, aliquoted, and stored at −80 °C. Samples were heat-inactivated for 1 h at 56 °C before running in the hemagglutination test (HAT) and enzyme-linked immunoassay (ELISA).

### Hemagglutination test (HAT)

The hemagglutination test (HAT)^16^ was used to investigate the SARS-CoV-2 specific antibodies to the RBD of the ancestral Wuhan-like, delta/B1.617.2 (L452R, T478K) and omicron BA.2 variants using codon optimised IH4-RBD sequence (see sequence alignment in Supplementary Fig. S2). Three additional mutations outside the antibody epitopes, Y365F, F392W and V395I, were included in the BA.2 RBD sequence to improve yield and stability (Supplementary Fig. S2). Sera were double diluted from 1:40 to 1:640 with 120ng/well of IH4-RBDs and equal volume of human 0-negative blood (∼1% v/v in PBS). Negative (PBS) and positive (EY6A) controls were included in each run. Red blood cells were allowed to settle for 1 h and positive wells agglutinated red blood cells. The HAT titre is defined as the last well in which a teardrop did not form.

### ELISA

The SARS-CoV-2 spike protein from the Wuhan virus was purified in house and used as coating antigen in the ELISA to detect spike specific IgG antibodies as previously described.^9^

### Statistical analysis

Data analysis and visualisation were performed in R version 4.1.3 (R Foundation for Statistical Computing, Vienna, Austria) and GraphPad Prism version 9.5.0 (USA). Illustrations were created with BioRender (Supplementary Fig. S1).

Questions about fatigue, concentration, and memory problems (from the Chalder questionnaire) and depression, originally assigned four categories in questionnaires 3- and 8-months post-infection (0 = less/better than usual, 1 = not more/worse than usual, 2 = more/worse than usual and 3 = much more/worse than usual), were converted to binary categories (0–1 vs 2–3).

**Fig. 1:**
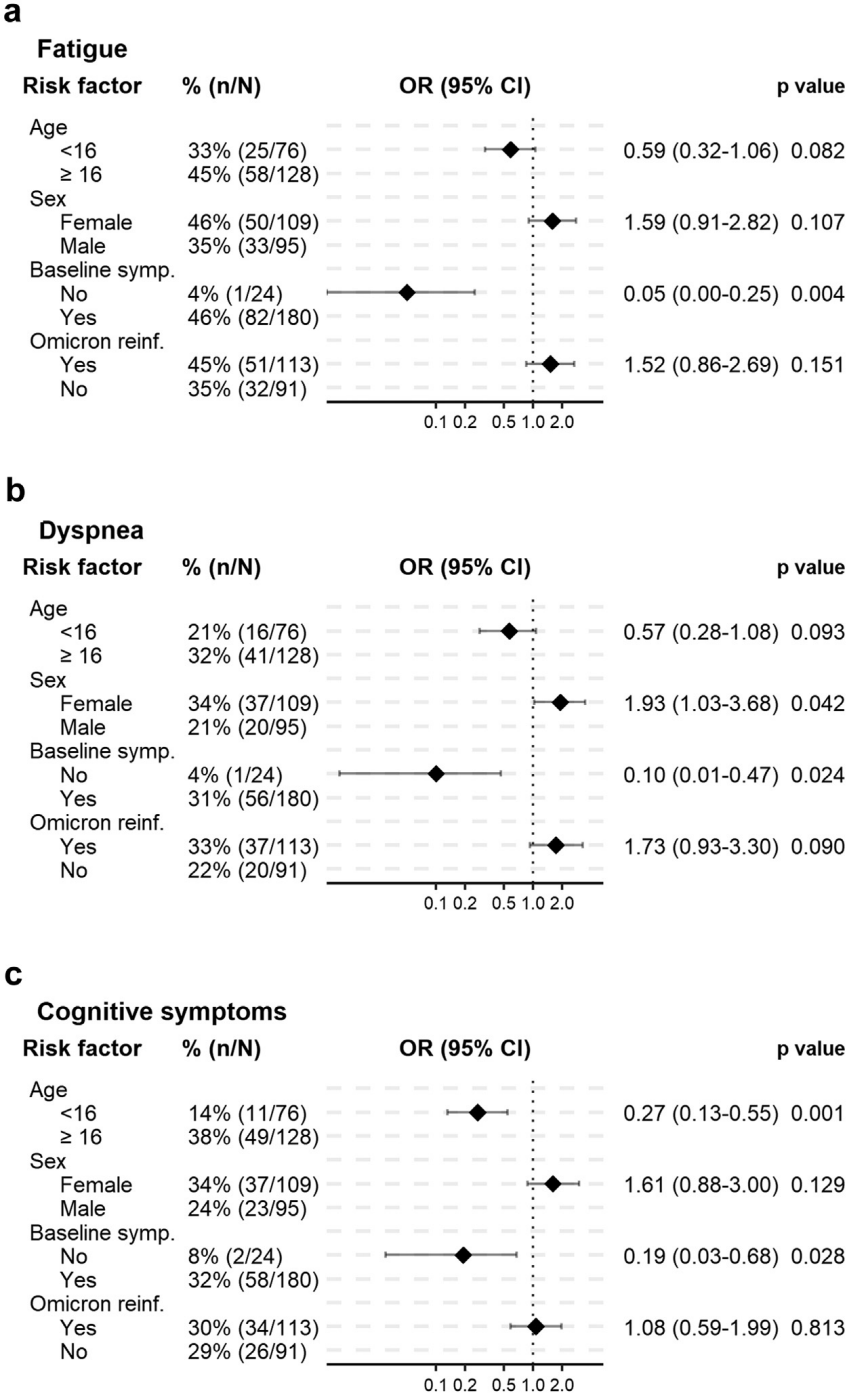
**Risk factors for long-term symptoms 8 months post delta infection.** Forest plots show odds ratios (OR) with 95% confidence intervals (CIs) and p-values calculated by univariable binary logistic regression models using relevant symptoms. The outcomes are **a** fatigue, **b** dyspnoea and **c** cognitive symptoms at 8 months and relevant risk factors are age (<16 or ≥16 years), sex, symptoms at baseline and omicron reinfection status. The number and percentage (n/N, %) of individuals in each risk factor are shown with the relevant outcomes in the table and forest plot.

**Fig. 2:**
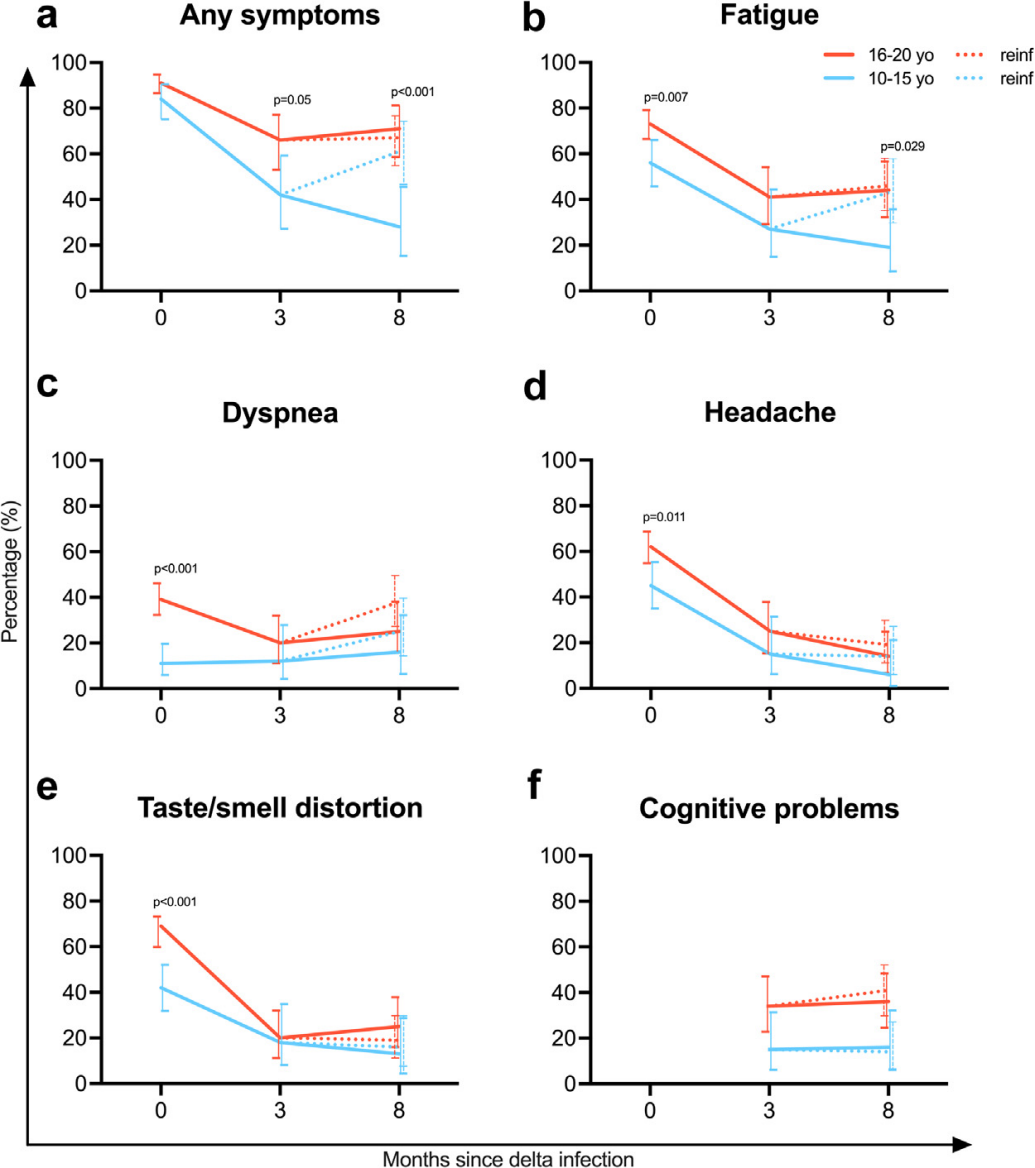
**Dynamics of long-term symptoms after delta infection by age group and omicron reinfection status.** Symptoms were recorded 0- (acute), 3- and 8-months post-infection. The percentage of symptoms after delta infection are shown by continuous lines for participants 10–15 (orange) and 16–20 (light blue) years, **a** any symptom, **b** fatigue, **c** dyspnoea, **d** headache, **e** taste/smell distortion and **f** cognitive impairment. The dashed line indicates the percentage of symptoms reported at 8 months by omicron reinfected individuals. Crude risk differences were calculated between age groups after delta infection.

The 95% CI in Fig. 1 and Table 1 were calculated by applying prop. test function in R with default settings (null hypothesis stating the two groups had the same proportions and two-sided Z test with Yates' continuity correction and with Wilson's score method). In Fig. 2, the percentages of symptoms are shown with 95% confidence intervals (CI) for proportions in this figure were calculated by Wald interval. Crude risk differences were calculated as an excess percentage of symptoms in one group compared to another group, and significant differences are shown by p values in the figure.

**Table 1:** Predictors for long-term symptoms 8 months post delta infection.

	N	OR (CI) P Unadjusted	OR (CI) P Adjusted
**Any persisting symptoms**			
Female sex	204	1.48 (0.84–2.61) 0.173	1.22 (0.66–2.23) 0.524
Age ≥16 years	204	2.44 (1.37–4.41) 0.003	2.33 (1.28–4.28) 0.006
Reinfection	204	1.43 (0.81–2.53) 0.214	1.41 (0.78–2.56) 0.254
Asymptomatic at baseline	204	0.28 (0.11–0.66) 0.005	0.35 (0.13–0.88) 0.029
**Dyspnoea**			
Female sex	204	1.93 (1.03–3.68) 0.042	1.61 (0.83–3.18) 0.161
Age ≥16 years	204	1.77 (0.92–3.51) 0.093	1.3 (0.63–2.71) 0.484
Reinfection	204	1.73 (0.93–3.3) 0.090	1.67 (0.86–3.3) 0.135
Dyspnoea baseline	204	4.21 (2.19–8.22) <0.001	3.63 (1.82–7.34) <0.001
**Cognitive symptoms**^a^			
Female sex	204	1.61 (0.88–3) 0.129	1.37 (0.72–2.65) 0.338
Age ≥16 years	204	3.67 (1.82–7.95) 0.001	3.36 (1.64–7.37) 0.001
Reinfection	204	1.8 (0.59–1.99) 0.813	1.05 (0.55–2.01) 0.881
Headache baseline	204	2.48 (1.33–4.76) 0.005	2.09 (1.08–4.12) 0.030
**Neurological symptoms**^b^			
Female sex	204	1.33 (0.62–2.92) 0.464	1.14 (0.52–2.56) 0.750
Age ≥16 years	204	2.97 (1.23–8.32) 0.023	2.72 (1.11–7.67) 0.039
Reinfection	204	1.4 (0.49–2.26) 0.915	1.02 (0.47–2.26) 0.962
Headache baseline	204	2.2 (1.01–5.11) 0.055	1.92 (0.85–4.58) 0.125
**Fatigue**			
Female sex	204	1.59 (0.91–2.82) 0.107	1.47 (0.82–2.66) 0.200
Age ≥16 years	204	1.69 (0.94–3.08) 0.082	1.5 (0.81–2.8) 0.202
Reinfection	204	1.52 (0.86–2.69) 0.151	1.43 (0.79–2.61) 0.233
Fatigue baseline	204	3.16 (1.67–6.21) 0.001	2.82 (1.47–5.62) 0.002

Age was used as a categorical variable to compare symptom prevalence in adolescents ≥16 years to children <16 years as a reference. Associated factors were reported as odds ratios (OR) with 95% confidence intervals (CIs) and p-values. In the multivariable analysis, adjustment was done for factors listed as predictors in the table.

a Cognitive symptoms include memory and concentration difficulties.

b Neurological symptoms include numbness, dizziness and sleeping problems.

Univariable and multivariable binomial logistic regression were used for analyses of binary outcome variables. The variables were selected both based on a priori hypothesis as explained above and suspicion of confounding (especially age and COVID vaccination). Variables were evaluated by a direct acyclic graph and selected based on multiple published studies showing that age, sex, baseline symptoms and vaccination affect long-term symptoms. The linearity assumption is checked both for univariable and multivariable analysis by using Box–Tidwell test (using car package with boxTidwell function). All of the interactions were insignificant.

**Fig. 3:**
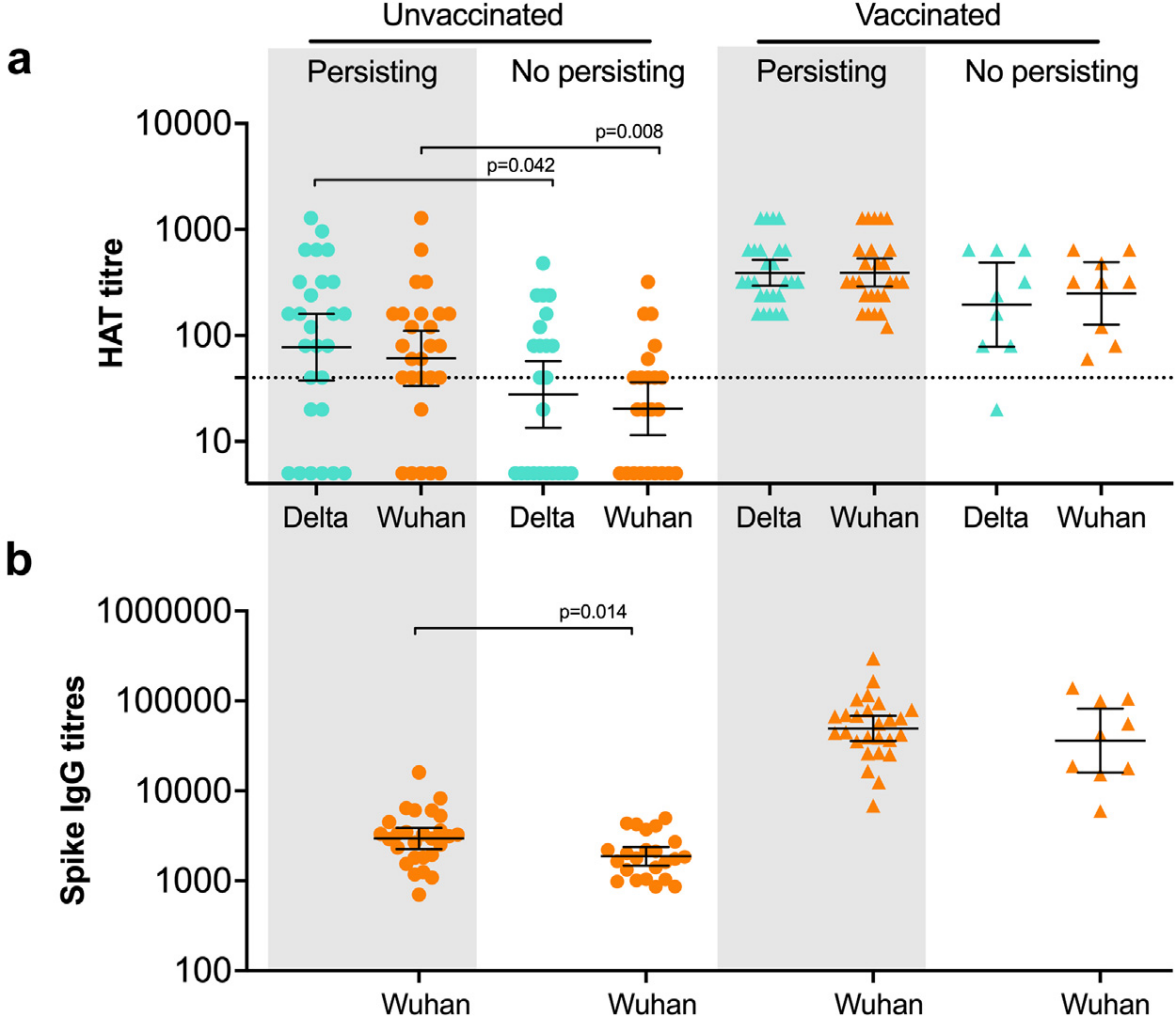
**The relationship between SARS-CoV-2 antibodies and persisting symptoms**. Hemagglutination test (HAT)-specific and binding (spike IgG) antibodies and persisting symptoms 3 months post-infection. The geometric mean antibody titres (GMT) with 95% confidence intervals are shown in black. **a** Delta (turquoise) and Wuhan (orange) antibodies and **b** Wuhan spike specific IgG antibody titres (orange) in individuals with persisting (grey background) and no persisting symptoms (white background). Vaccination status is shown by circles (unvaccinated) and triangles (vaccinated). Each symbol represents one individual. The dotted line indicates a positive test (HAT titre of ≥40), associated with neutralising antibodies. Negative values were assigned a value of 5. The differences between antibody titres in groups with persisting and no persisting symptoms were compared by the Mann Whitney U test.

**Fig. 4:**
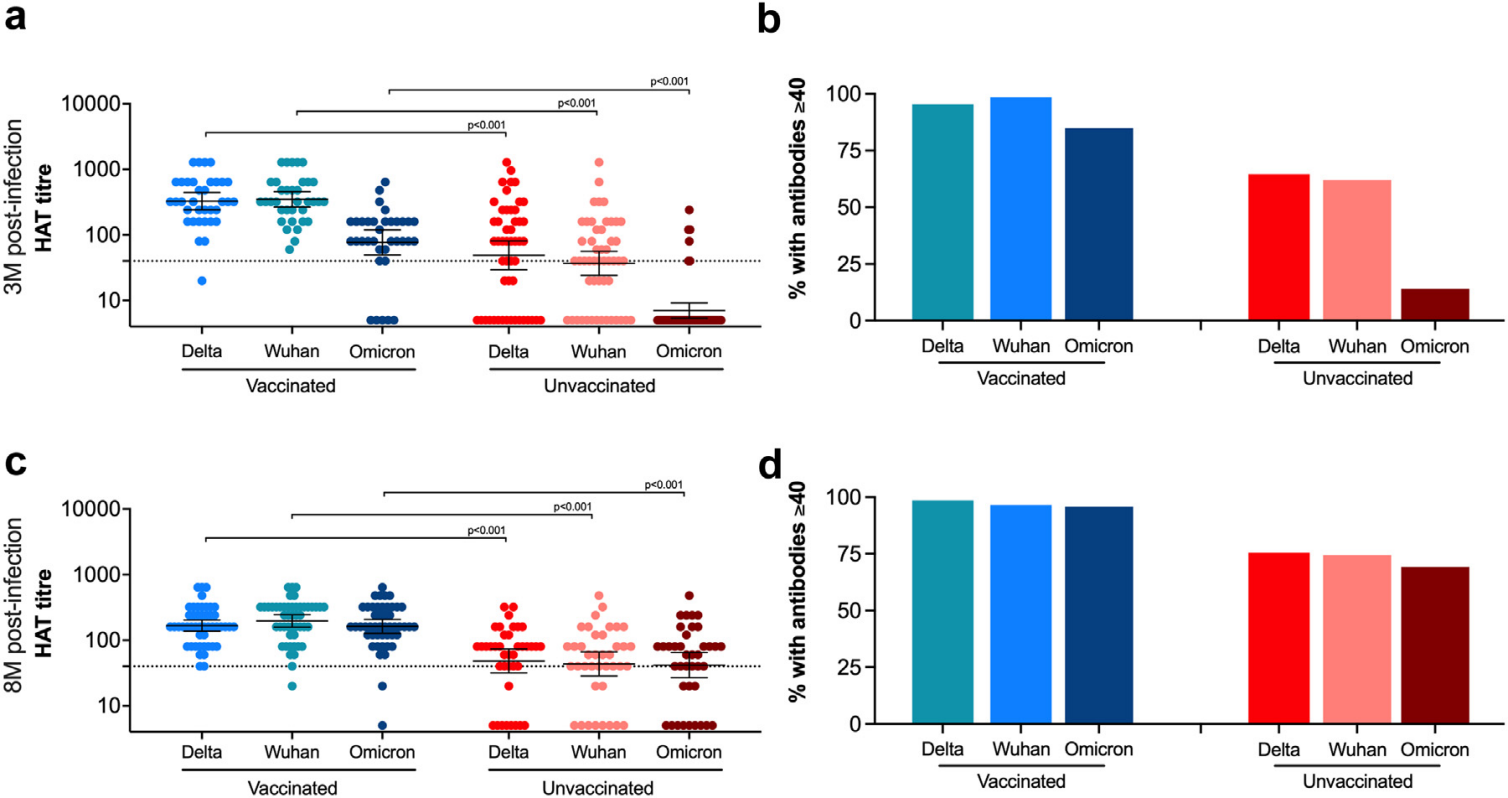
**Antibody titres in vaccinated and unvaccinated individuals**. Hemagglutination test (HAT)-specific antibodies to the delta, Wuhan and omicron SARS-CoV-2 receptor binding domain (RBD), HAT titres (**a,c)** and percentage (%) with titres ≥40 (**b,d**). Sera were collected at 3 (**a,b)** and 8 months **(c,d)** post delta infection. At 3 months, individuals were divided based on pre delta infection vaccination status: vaccinated (blue, n = 35, sampled mean 76.1 days) and unvaccinated (red, n = 53, sampled mean 87.9 days). At 8 months, individuals who had never been previously COVID vaccinated were defined as unvaccinated (red, n = 39, sampled mean 147.0 days), and individuals with any previous vaccination, n = 48 (blue, n = 48, sampled mean 95.4 days). At 8 months, the percentage of reinfected individuals was comparable (54.5% in vaccinated and 66.7% in unvaccinated). Each individual is indicated by a circle. The dotted line indicates a positive test (HAT titre of ≥40), and negative values were assigned a value of 5. The geometric mean titres (GMT) with 95% confidence intervals are shown in black by horizontal lines. Vaccinated individuals were compared to unvaccinated by a Mann Whitney U test for each variant. Significant differences were maintained after adjusting for days since infection or vaccination at both time points.

IgG and HAT antibody titres in Figs. 3 and 4 and Table 1, Supplementary Tables S3 and S4 were log(10)-transformed to correct for skewness of distribution. Mann–Whitney U test was used to compare untransformed continuous variables in Figs. 3 and 4.

### Role of funding sources

Funders had no role in study design, data collection, data analyses, interpretation, or writing of report.

## Results

### Cohort descriptive

The cohort consisted of 276 children and adolescents (BMI range 15.8–30.1), with mean age of 16.5 years (range 10–20 years), 54% were females (Supplementary Table S1). The most common comorbidities were seasonal allergies (12%) and asthma (9%). All participants had mild, self-limiting delta SARS-CoV-2 infection, not requiring hospitalisation.

A total of 103 participants (37%), all 16 years or older (mean age 19.0 years), had received their priming COVID mRNA-vaccine dose (primarily Comirnaty (BioNTech/Pfizer)) in June and July 2021, on average 1.5 months prior to the positive RT-PCR test (Supplementary Table S1, Supplementary Fig. S1). Based on the difference in vaccine recommendations, the cohort was divided into groups over and under 16 years.

### Acute symptoms impacted persisting and long-term symptoms

Participants were RT-PCR tested for SARS-CoV-2 mainly due to acute symptoms (89%) (Supplementary Table S1). All participants with available serum samples 3 months post-infection (n = 88) had spike-specific IgG antibodies (end point titres >485), and seropositivity was 85% for HAT antibodies (titres ≥40) (surrogate neutralisation titres). The most common acute symptoms in symptomatic cases were fatigue (68%), distorted taste and smell (59%), fever (58%), cough (57%) and headache (57%) (Supplementary Table S2). The median duration of acute symptoms (n = 221) was 4 and 7 days for children and adolescents, respectively. Twenty-five participants had ongoing symptoms after the acute phase.

Eleven percent (30/276) of the participants had asymptomatic acute infection and were RT-PCR tested mostly due to close contact with a SARS-CoV-2 positive. Cases who were asymptomatic at baseline were less likely to have symptoms both at 3 (OR 0.13, 95% CI 0.02–0.55) and 8 (OR 0.28, CI 95% 0.11–0.66) months post-infection (Table 1, Fig. 1, Supplementary Table S3). Individuals reporting dyspnoea or fatigue during acute illness, were more likely to report these symptoms long-term at 8 months (OR 4.21, 95% CI 2.19–8.22 and OR 3.16, 95% CI 1.67–6.21, respectively) (Table 1). Experiencing headache during the acute illness was associated with cognitive dysfunction (impaired memory and concentration) (OR 2.48, 95% CI 1.33–4.76) 8 months post-acute infection (Table 1).

Young age (<16 years) was associated with reporting fewer symptoms. Adolescents (16–20 years) more frequently experienced acute symptoms (fatigue, dyspnoea, headache and taste-smell distortion) and long-term symptoms (“any symptoms”, fatigue) (Fig. 2). In the group eligible for vaccination (adolescents ≥16 years), there was no association between vaccination and frequency of symptoms (Supplementary Fig. S3).

At 3 months post delta infection (median 104 days, IQR 62–109 days), we followed a subgroup of 89 participants with questionnaires and serum samples (n = 88). We found that 56% of the cohort reported persisting symptoms at 3 months, although with a lower symptom burden than during the acute phase (mean number of symptoms 1.8 versus 4.0 in the acute phase). The most prominent symptoms were fatigue (36%), impaired concentration (27%) and headache (21%), especially in adolescents (Supplementary Table S2). Older age (≥16 years) was associated with persisting symptoms (Supplementary Table S3).

### Convalescent antibody levels were associated with persisting symptoms

In unvaccinated participants, persisting symptoms at 3 months were associated with higher delta (p = 0.042) and Wuhan-specific HAT antibody titres (p = 0.008) (Fig. 3a) as well as Wuhan-specific spike IgG (p = 0.048) (Mann–Whitney U test) (Fig. 3b). Convalescent IgG spike antibodies were significantly correlated with persisting symptoms (Supplementary Table S3). Overall, vaccinated individuals had significantly higher convalescent antibodies (Fig. 4) than unvaccinated subjects, but had no difference in antibody titres in respect to persisting symptoms (Fig. 3).

### Long-term symptoms post delta infection

When assessing long-term symptoms after delta infection, omicron reinfected participants were excluded. At the 8-month follow-up (median 245, IQR 205–254 days), the three most prevalent symptoms were fatigue (35%), cognitive problems (29%) and dyspnoea (22%) (Supplementary Table S2). Children <16 years reported less frequent symptoms, while adolescents had unchanged proportions of fatigue, dyspnoea, neurological or cognitive symptoms (Supplementary Table S2, Fig. 2). While long-term dyspnoea and fatigue were not associated with age, composite and specific symptoms such as cognitive dysfunction were associated with age (OR 3.67, 95% CI 1.82–7.95) (Table 1, Fig. 1). Females more frequently had long-term dyspnoea (OR 1.93, 95% CI 1.03-3.68) but no other specific symptoms (Fig. 1b). Depression was recorded only at 8-month follow-up, and 15% of our cohort reported feeling more sad or depressed than usual, of whom 75% were female (Supplementary Table S2). At 8 months, the data did not show evidence of an association between vaccination status and symptom prevalence in participants over 16 years, but the number of unvaccinated participants was low (n = 22) (Supplementary Table S4).

During our follow-up, we recorded symptom-impact on daily functioning, specifically school and/or work absenteeism and the ability to participate in extracurricular activities. At the 8-month follow-up, only 9 asymptomatic individuals (16%) were absent from work and/or extracurricular activities. In the individuals reporting absence, we found that 60% reported fatigue, compared to 33% fatigue in those without absence (OR 3.1, 95% CI 1.6–5.9) (Supplementary Table S5).

### The impact of omicron on long-term symptoms

The omicron reinfected group reported more long-term respiratory and systemic symptoms than those not reinfected, who had only experienced delta infection (Fig. 1, Supplementary Table S2), particularly in participants under 16 years (Fig. 2). There was, however, no difference in symptoms such as taste/smell, cognitive and neurological symptoms compared to delta infection alone (Fig. 1, Supplementary Table S1). After delta infection, the adolescent group had more long-term symptoms compared to children. Interestingly, adolescents reported no change in symptoms after omicron reinfection. This effect reduced the overall difference in symptoms reported by children and adolescents at 8 months (Fig. 2).

## Discussion

In our prospective paediatric study, we showed that older age, having acute symptoms, and higher antibody titres are associated with long-term symptoms after SARS-CoV-2 delta infection. The association between antibody titres and long-term symptoms previously described in adults,^8^^,^^9^^,^^15^ can in this study be extended to children. Hence, an immune dysfunction may also be involved in maintaining symptoms in children,^17^ as observed in adolescents after other viral infections.

The three most common long-term symptoms after delta infection in our cohort were fatigue, dyspnoea and cognitive impairment, with higher frequencies in adolescents than in children. These three were recently confirmed as the common clusters of long-term COVID symptoms,^18^ and frequently reported as persisting paediatric symptoms.^3^^,^^11^^,^^19^^,^^20^ We observed that adolescents more frequently reported persistent and long-term symptoms than children, confirming the association between symptoms and age post-puberty,^5^ despite high vaccination rates in adolescents. When comparing groups of vaccinated and unvaccinated adolescents, we found no significant differences in reported acute and persistent symptoms, although the unvaccinated group at 3 months was small. Many studies have found a reduced risk of long COVID symptoms after vaccination in adults,^14^ and this is yet to be confirmed in younger age groups.

The course of long COVID can be fluctuating and protracted with worsening symptoms over time.^8^^,^^21^^,^^22^ When investigating the individuals’ symptoms over time, we observed that most symptoms improved, while others worsened, and some reported new symptoms during the study period. This fluctuation of symptoms is an essential characteristic of post COVID-19 condition, as defined by the WHO, and has been documented by other studies.^3^ Furthermore, the post COVID-19 condition comprehend any impact on daily life, such as absence from school, work, or extracurricular activities. In 40% (49/124) of the young people in our study, symptoms were associated with abstenteeism, compared to only 11% absence in the asymptomatic group.

Our study did not detect a higher frequency of depression or sadness than the Norwegian national average in 13–19-year-olds.^23^ A recent study in children and adolescents reported that mental health long-term symptoms were more prevalent in cases compared to controls.^24^ COVID-associated anxiety and mood disorders have been found to gradually subside over time.^25^ In agreement with this, our 8 months data cannot confirm an association between COVID infection and long-term depressive symptoms. A recent large Norwegian study found limited increase in healthcare utilisation among adolescents 6 months after COVID-19.^26^ However, concentration and memory problems may go unnoticed by the health care system due to their non-specific nature.

We confirm that acute symptoms and increasing age are factors associated with persisting symptoms.^3^ A paediatric Australian study, with a very low median age of 3 years and high proportion of asymptomatic infections, found that children had fully recovered 3–6 months post-Wuhan infection.^27^ In our cohort, children were more often asymptomatic than adolescents. Although most symptoms generally improved over time, 28% of children and 71% of adolescents in our cohort reported one or more long-term symptoms at 8 months. However, our study design does not allow inference about the contribution of COVID-19 to these symptoms, and any comparison should solely be made between these two age groups.

Our study is unique in combining immunological results with detailed long-term symptoms, largely based on personal interviews. Further strengths are long-term follow-up for 8 months after delta infection, including omicron reinfection rates. In contrast to other larger epidemiological studies, we have prospectively recruited participants shortly after infection, ensuring limited recall bias of acute symptoms. Our dropout rate was low, with 74% responding at 8 months. A strength of the study was that children and adolescents could be compared in the same geographic area, time period, with identical variant exposure, enabling exploration of the relative differences in long-term symptomatology.

Caveats to our study are the lack of a COVID negative control group which may overestimate the incidence of persisting symptoms attributable to SARS-CoV-2 infection, although our study was not designed to assess prevalence. Nevertheless, our findings are similar to studies with case control design.^24^^,^^28^ Inclusion in this study was after delta infection, and we could therefore not control for pre-infection physical and mental health. Methodological limitations to the study are potential unmeasured confounding factors, and indirect data collection by parents of participants <16 years, although these children actively participated in the interviews. A modest sample size increases the risk of sparse data bias as evident by large effect size and confidence limits. The ongoing vaccination campaign during the study period skewed the vaccination coverage exclusively to adolescents, with only a few adolescents (n = 22/187) unvaccinated at 8 months, limiting interpretation of the impact of vaccination on long-term symptoms. Omicron reinfection increased the heterogeneity and hampered the long-term evaluation of delta-only infection. We were not able to investigate the relationship between antibody titres and 8 months symptoms due to antibody boosting after vaccinations and omicron reinfections leading to small groups available for comparison.

With emerging variants, hybrid immunity and fluctuating nature of post COVID-19 syndrome, it is difficult to diagnose and study paediatric long-term symptoms during an evolving pandemic. Our findings emphasise the importance of reducing the COVID-19 burden in young people and may shed light on the underlying pathophysiology of this syndrome.

## Contributors

N.L. and R.J.C. designed the study. N.U.E, K.K, E.B.F, and K.H recruited the participants and followed them up. A.I. supplemented and distributed the electronic questionnaires. P.R. developed the HAT reagents. N.U.E and J.S.O conducted laboratory analysis. T. Ö, B.B, C.J.B, E.B.F., R.J.C, N.L. and N.U.E. analysed the data and conceptualised the analyses. N.U.E, N.L and R.J.C wrote the first version of the manuscript, and verified the underlying data. B.B. and A.I. critically revised the manuscript. Members of the Bergen COVID-19 research group contributed to the study follow-up, data collection, and laboratory assays. All authors read and approved the final version of the manuscript.

## Data sharing statement

The R code used to generate all results in this paper is publicly available on GitHub https://github.com/turkulerc/DeltaKids. Source data are available upon reasonable request to the corresponding author.

## Declaration of interests

All authors declare no conflict of interest.

## Appendix A Supplementary data

Supplementary Figs. S1–S3 and Tables S1–S5

Consortia authors
