# Supplementary material for: Post COVID-19 condition after delta infection and omicron reinfection in children and adolescents
Source: eBioMedicine. 2023 May 5;92:104599. doi: 10.1016/j.ebiom.2023.104599 (PMC10166589; doi:10.1016/j.ebiom.2023.104599)
Supplement: Supplementary Figs. S1–S3 and Tables S1–S5 [file mmc1.docx]

**Supplementary material**

Table of contents

SUPPlementary table 1. Cohort demographics 2

Supplementary table 2. Symptoms in children and adolescents up to 8 months post delta infection 3

Supplementary table 3. Predictors for persisting symptoms 3 months post delta infection 4

Supplementary table 4. Predictors for long-term symptoms 8 months post delta infection in adolescents (≥16 years old) 5

Supplementary table 5. The impact of long-term symptoms on absteinteeism from work and extracurricular activities 8 months post delta infection 6

Supplementart figure 1. Study design 7

Supplementary figure 2. Sequence alignment in the sars-cov-2 receptor binding domain (RBD) regions of wuhan, delta and omicron (BA.1 and BA.2) 8

SUPPLEMENTARY FIGURE 3. ACUTE AND PERSISTENT SYMPTOMS IN VACCINATED AND UNVACCINATED INDIVIDUAL 9

**Supplementary Table 1. Cohort demographics.**

|  | | | **All** | **10-15 years old** | **16-20 years old** |
| --- | --- | --- | --- | --- | --- |
| **Number (n)** | | | 276 | 89 | 187 |
| **Mean age, years (SD)** | | | 16.5 (2.8) | 13.2 (1.7) | 18.1 (1.5) |
| **Sex (%)** | | |  |  |  |
|  | Female | | 150 (54) | 44 (49) | 106 (57) |
|  | Male | | 126 (46) | 45 (51) | 81 (43) |
| Mean body mass index, BMI (SD) | | | 21.6 (2.6) | 19.9 (2.3) | 22.3 (2.5) |
| **Vaccination^a^** | | | 103 (37%) | 0^b^ | 103 (55%) |
|  | | 1^st^ dose | 98/103 (95%) | 0 | 98/103 (95%) |
|  | | 2^nd^ dose | 5 (5%) | 0 | 5 (5%) |
|  | | Comirnaty (BioNTech/Pfizer) | 97 (94%) | 0 | 97 (94%) |
|  | | Spikevax (Moderna) | 6 (6%) | 0 | 6 (6%) |
| **Comorbidities** | | | 47/193 (24%) | 15 (22%) | 32 (26%) |
|  | | Seasonal allergies | 24 (12%) | 5 (7%) | 19 (15%) |
|  | | Asthma | 17 (9%) | 7 (10%) | 10 (8%) |
|  | | Gastrointestinal disease | 4 (2%) | 1 (2%) | 3 (2%) |
|  | | Neurological disease | 4 (2%) | 1 (2%) | 3 (2%) |
|  | | Chronic cardiovascular disease | 1 (1%) | 1 (2%) | 0 (0) |
|  | | Rheumatological disease | 1 (1%) | 1 (2%) | 0 (0) |
| **Medication** | | | 47/191 (25%) | 15/67 (22%) | 32/124 (26%) |
|  | | Antihistamine | 6 (3%) | 2 (3%) | 4 (3%) |
|  | | Contraceptives | 5 (3%) | 0 (0) | 5 (4%) |
|  | | Inhalation steroids | 3 (2%) | 2 (3%) | 1 (1%) |
|  | | Immunosuppression | 3 (2%) | 1 (2%) | 2 (2%) |
|  | | Stimulants (ADHD medication) | 2 (1%) | 0 (0) | 2 (2%) |
|  | | Hormone substitution | 1 (1%) | 0 (0) | 1 (1%) |
|  | | Migraine medication | 2 (1%) | 1 (2%) | 1 (1%) |
|  | | Preventative cardiovascular medication | 1 (1%) | 0 (0) | 1 (1%) |
|  | | Other^c^ | 24 (13%) | 9 (13%) | 15 (12%) |

^a^Vaccination prior to delta infection

^b^Vaccination not recommended at inclusion time point

^c^Missing specific information

**Supplementary Table 2. Symptoms in children and adolescents up to 8 months post delta infection.**

|  | | **Delta** | | | | | | | | | **Omicron reinfection*** | | |
| --- | --- | --- | --- | --- | --- | --- | --- | --- | --- | --- | --- | --- | --- |
|  | | **Acute phase symptoms** | | | **3 months post-infection** | | | **8 months post-infection** | | | **2 months post-reinfection** | | |
| **Age (years)** | | All | 10-15 | 16-20 | All | 10-15 | 16-20 | All | 10-15 | 16-20 | All | 10-15 | 16-20 |
| **Number** | | 276 | 89 | 187 | 89 | 33 | 56 | 91 | 32 | 59 | 113 | 44 | 69 |
| Asymptomatic | | 30 (11%) | 14 (16%) | 16 (9%) | 40 (45%) | 19 (58%) | 19 (34%) | 40 (44%) | 23 (72%) | 17 (29%) | 40 (35%) | 17 (39%) | 23 (33%) |
| Any symptoms | | 246 (89%) | 75 (84%) | 171 (91%) | 51 (57%) | 14 (42%) | 37 (66%) | 51 (56%) | 9 (28%) | 42 (71%) | 73 (65%) | 27 (61%) | 46 (67%) |
| Any general symptoms | | 225 (82%) | 68 (76%) | 157 (84%) | 39 (44%) | 11 (33%) | 28 (50%) | 36 (40%) | 6 (19%) | 30 (51%) | 55 (49%) | 20 (45%) | 35 (51%) |
|  | Fever | 161 (58%) | 49 (55%) | 112 (60%) | 5 (6%) | 1 (3%) | 4 (7%) | 1 (1%) | 0 (0) | 1 (2%) | 8 (7%) | 2 (5%) | 6 (9%) |
|  | Fatigue | 187 (68%) | 50 (56%) | 137 (73%) | 32 (36%) | 9 (27%) | 23 (41%) | 32 (35%) | 6 (19%) | 26 (44%) | 51 (45%) | 19 (43%) | 32 (46%) |
|  | Headache | 156 (57%) | 40 (45%) | 116 (62%) | 19 (21%) | 5 (15%) | 14 (25%) | 10 (11%) | 2 (6%) | 8 (14%) | 19 (17%) | 6 (14%) | 13 (19%) |
|  | Gastro-intestinal symptoms | 45 (16%) | 14 (16%) | 31 (17%) | 7 (8%) | 2 (6%) | 5 (9%) | 8 (9%) | 0 (0) | 8 (14%) | 4 (4%) | 1 (2%) | 3  (4%) |
|  | Muscle/joint pain | 81 (29%) | 17 (19%) | 64 (34%) | - | - | - | 2 (2%) | 0 (0) | 2 (3%) | 8 (7%) | 2 (5%) | 6  (9%) |
|  | Palpitations | - | - | - | 7 (8%) | 2 (6%) | 5 (9%) | 5 (5%) | 1 (3%) | 4 (7%) | 5 (4%) | 1 (2%) | 4  (6%) |
| Any respiratory symptoms | | 216 (78%) | 68 (76%) | 148 (79%) | 19 (21%) | 7 (21%) | 12 (21%) | 29 (32%) | 5 (16%) | 24 (41%) | 59 (52%) | 20 (45%) | 39 (57%) |
|  | Dyspnoea | 83 (30%) | 10 (11%) | 73 (39%) | 15 (17%) | 4 (12%) | 11 (20%) | 20 (22%) | 5 (16%) | 15 (25%) | 37 (33%) | 11 (25%) | 26 (38%) |
|  | Cough | 158 (57%) | 38 (43%) | 120 (64%) | - | - | - | 0 (0) | 0 (0) | 0 (0) | 13 (12%) | 4 (9%) | 9 (13%) |
|  | Congested nose/sore throat | 64 (23%) | 26 (29%) | 38 (20%) | 6 (7%) | 5 (15%) | 1 (2%) | 4 (4%) | 1 (3%) | 3 (5%) | 8 (7%) | 5 (11%) | 3 (4%) |
|  | Chest pain | - | - | - | - | - | - | 6 (7%) | 2 (6%) | 4 (7%) | 6 (5%) | 1 (2%) | 5 (7%) |
| Smell/taste | | 162 (59%) | 37 (42%) | 125 (69%) | 17 (19%) | 6 (18%) | 11 (20%) | 19 (21%) | 4 (13%) | 15 (25%) | 20 (18%) | 7 (16%) | 13 (19%) |
| Any neurological symptoms | | - | - | - | 19 (21%) | 8 (24%) | 11 (20%) | 14 (15%) | 1 (3%) | 13 (22%) | 18 (16%) | 5 (11%) | 13 (19%) |
|  | Numbness | - | - | - | 0 (0) | 0 (0) | 0 (0) | 3 (3%) | 0 (0) | 3 (5%) | 5 (4%) | 1 (2%) | 4 (6%) |
|  | Dizziness | - | - | - | 12 (14%) | 7 (21%) | 5 (9%) | 9 (10%) | 1 (3%) | 9 (14%) | 12 (11%) | 3 (7%) | 9 (13%) |
|  | Sleeping problems | - | - | - | 10 (11%) | 2 (6%) | 8 (14%) | 7 (8%) | 0 (0) | 7 (12%) | 8 (7%) | 2 (5%) | 6 (9%) |
| Depression | | - | - | - | - | - | - | 15 (16%) | 4 (13%) | 11 (19%) | 17 (15%) | 2 (5%) | 15 (22%) |
| Any cognitive symptoms | | - | - | - | 24 (27%) | 5 (15%) | 19 (34%) | 26 (29%) | 5 (16%) | 21 (36%) | 34 (30%) | 6 (14%) | 28 (41%) |
|  | Impaired memory | - | - | - | 14 (16%) | 3 (9%) | 11 (20%) | 19 (21%) | 4 (13%) | 15 (25%) | 19 (17%) | 3 (7%) | 16 (23%) |
|  | Impaired concentration | - | - | - | 21 (24%) | 5 (15%) | 16 (29%) | 20 (22%) | 5 (16%) | 15 (25%) | 25 (22%) | 4 (9%) | 21 (30%) |
| Other problems | | 17 (6%) | 4 (5%) | 13 (7%) | 11 (12%) | 1 (3%) | 10 (18%) | 4 (4%) | 1 (3%) | 3 (5%) | 9 (8%) | 3 (7%) | 6 (9%) |

Data presented as numbers (percentage, %). *n=2 individuals were delta reinfected

**Supplementary Table 3. Predictors for persisting symptoms 3 months post delta infection.**

#

|  | | N | OR (CI) P Unadjusted | OR (CI) P Adjusted |
| --- | --- | --- | --- | --- |
| Any persisting symptoms | |  |  |  |
|  | Female sex | 89 | 0.99 (0.42-2.3) 0.973 | 0.53 (0.18-1.51) 0.249 |
|  | Age ≥16 years | 89 | 2.64 (1.1-6.52) 0.031 | 1.7 (0.47-6.26) 0.416 |
|  | Asymptomatic baseline | 89 | 0.13 (0.02-0.55) 0.013 | 0.08 (0.01-0.45) 0.01 |
|  | Antibodies (IgG) Wuhan^#^ | 88 | 2.97 (1.57-6.04) 0.001 | 3.65 (1.53-9.83) 0.006 |
|  | Vaccination | 89 | 0.47 (0.18-1.15) 0.105 | 2.23 (0.55-10.04) 0.274 |

Age was used as a categorical variable to compare symptom prevalence in adolescents ≥16 years children <16 years as a reference. Associated factors were reported as odds ratios (OR) with 95% confidence intervals (CIs) and p-values. In the multivariable analysis, adjustment was done for factors listed as predictors in the table.

*Cognitive symptoms include memory and concentration difficulties.

^#^Spike IgG are log-transformed and used as a continuous, independent variable

# **Supplementary Table 4. Factors associated with long-term symptoms 8 months post delta infection in adolescents (≥16 years old)**

|  | N | Estimate (CI) P Unadjusted | Estimate (CI) P Adjusted |
| --- | --- | --- | --- |
| Any persisting symptoms |  |  |  |
| Female sex | 128 | 1.6 (0.75-3.41) 0.223 | 1.5 (0.66-3.38) 0.331 |
| Age | 128 | - 1. 0.63-1.06) 0.131 | 0.77 (0.57-1.01) 0.064 |
| Reinfection | 128 | - 1. 0.38-1.71) 0.583 | 0.8 (0.37-1.73) 0.572 |
| Any symptoms at baseline | 128 | 1.29 (0.32-4.54) 0.703 | 1.32 (0.3-5.31) 0.697 |
| Vaccination | 128 | 1.67 (0.63-4.29) 0.286 | 1.98 (0.7-5.57) 0.191 |
| Dyspnoea |  |  |  |
| Female sex | 128 | - 1. 0.88-4.15) 0.107 | 1.59 (0.69-3.71) 0.227 |
| Age | 128 | 0.93 (0.72-1.2) 0.566 | - 1. (0.66-1.16) 0.366 |
| Reinfection | 128 | - 1. 0.84-3.86) 0.14 | 1.83 (0.81-4.22) 0.149 |
| Dyspnoea baseline | 128 | 4.35 (2-9.74) <0.001 | 4.23 (1.91-9.69) <0.001 |
| Vaccination | 128 | 1.31 (0.49-3.93) 0.6 | 1.34 (0.44-4.51) 0.62 |
| Cognitive symptoms* |  |  |  |
| Female sex | 128 | 1.93 (0.93-4.09) 0.079 | - 1. (0.72-3.55) 0.258 |
| Age | 128 | - 1. (0.6-0.99) 0.043 | 0.7 (0.53-0.91) 0.011 |
| Reinfection | 128 | 1.24 (0.6-2.55) 0.563 | - 1. (0.57-2.66) 0.605 |
| Headache baseline | 128 | 2.44 (1.16-5.33) 0.022 | 2.59 (1.16-6.04) 0.023 |
| Vaccination | 128 | 1.82 (0.69-5.41) 0.248 | 2.67 (0.9-8.98) 0.09 |
| Neurological symptoms^§^ |  |  |  |
| Female sex | 128 | 1.12 (0.47-2.73) 0.798 | 1.01 (0.4-2.57) 0.989 |
| Age | 128 | - 1. (0.61-1.11) 0.208 | 0.81 (0.59-1.09) 0.168 |
| Reinfection | 128 | 0.82 (0.34-1.96) 0.655 | 0.81 (0.34-1.97) 0.647 |
| Headache baseline | 128 | 1.43 (0.59-3.65) 0.432 | 1.52 (0.6-4.05) 0.387 |
| Vaccination | 128 | 1.18 (0.39-4.39) 0.785 | 1.53 (0.47-6.15) 0.511 |
| Fatigue |  |  |  |
| Female sex | 128 | 1.87 (0.92-3.84) 0.086 | 1.9 (0.88-4.17) 0.104 |
| Age | 128 | 0.84 (0.66-1.07) 0.158 | 0.74 (0.56-0.96) 0.027 |
| Reinfection | 128 | 1.1 (0.55-2.22) 0.794 | 1.07 (0.5-2.28) 0.866 |
| Fatigue baseline | 128 | 3.92 (1.68-10.08) 0.003 | 4.85 (1.98-13.09) 0.001 |
| Vaccination | 128 | 1.56 (0.62-4.2) 0.357 | 2.03 (0.72-6.16) 0.191 |

Age was used as a continuous variable. Associated factors were reported as odds ratios (OR) with 95% confidence intervals (CIs) and p-values. In the multivariable analysis, adjustment was done for factors listed as predictors in the table.

*Cognitive symptoms include memory and concentration impairment.

^$^Neurological symptoms include numbness, dizziness and sleeping problems.

# **Supplementary table 5: The impact of long-term symptoms on absenteeism from work and extracurricular activities at 8 months post delta infection.**

|  | **Absenteeism (n/N, %)** | | **OR (CI)** |
| --- | --- | --- | --- |
|  | No | Yes |  |
| **N** | 146 | 58 |  |
| **Age (years)** |  |  |  |
| <16 | 54 (37%) | 22 (38%) | 1.0 (0.5-2.09) |
| ≥16 | 92 (63%) | 36 (62%) |  |
| **Sex** |  |  |  |
| Female | 75 (51%) | 34 (59%) | 1.4 (0.7-2.5) |
| Male | 71 (49%) | 24 (41%) |  |
| **Asymptomatic** |  |  |  |
| Baseline | 21 (14%) | 3 (5%) | 0.3 (0.1-1.0) |
| 8 months | 71 (49%) | 9 (16%) | 0.2 (0.1-0.4) |
| **Symptoms 8M** |  |  |  |
| Fatigue | 48 (33%) | 35 (60%) | 3.1 (1.6-5.9) |
| Dyspnoea | 31 (21%) | 26 (45%) | 3.0 (1.6-5.8) |
| Cognitive symptoms | 41 (28%) | 19 (33%) | 1.2 (0.6-2.4) |
| Depression | 15 (10%) | 17 (29%) | 3.6 (1.6-8.0) |

**Supplementary figure 1. Study design.**
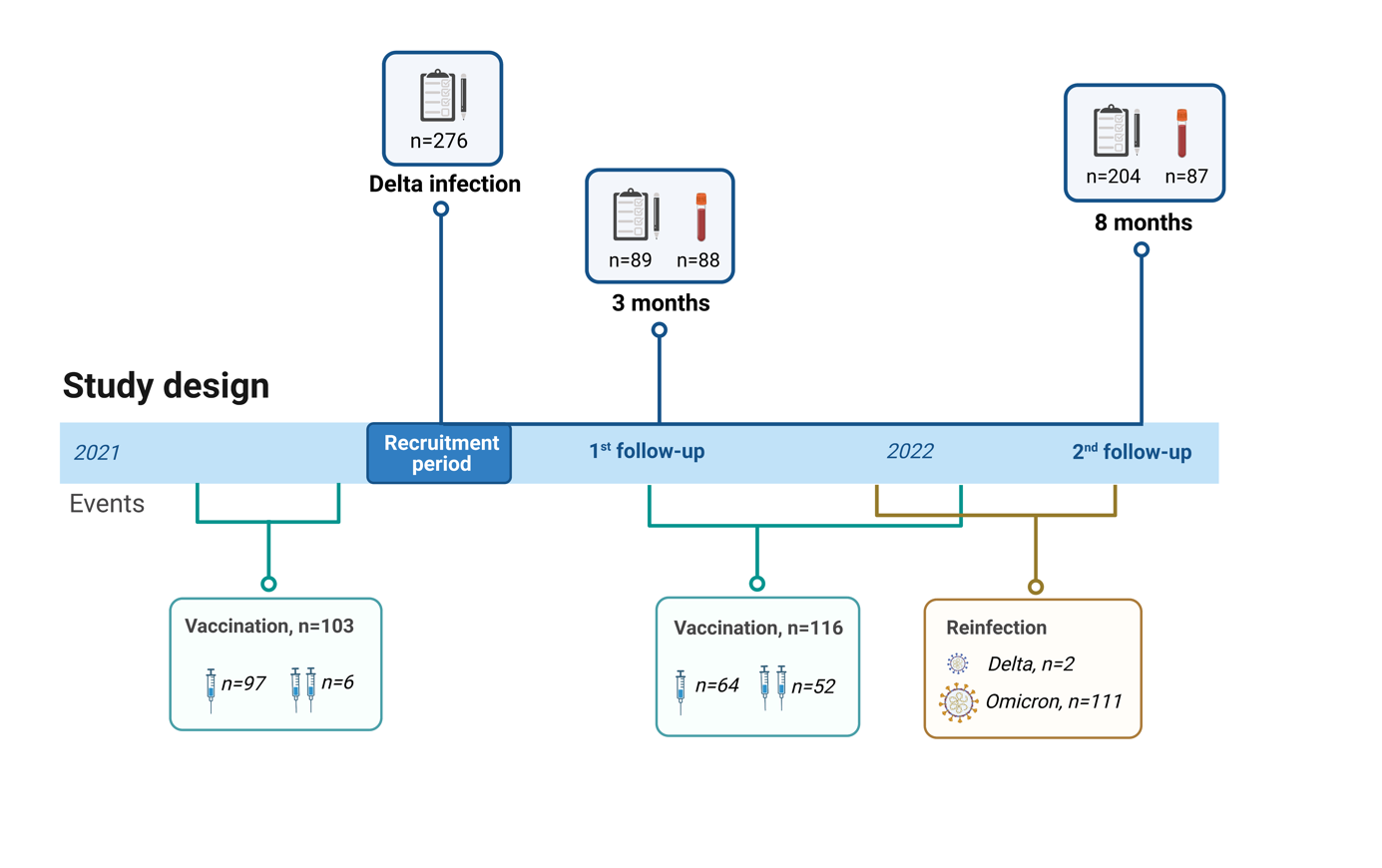


276 delta infected children and adolescents 10-20 years old were recruited after RT-PCR-confirmed SARS-CoV-2 infection, from August 1^st^ to September 16^th^, 2021. Questionnaires about acute, persisting, and long-term symptoms were answered at recruitment (baseline) (n=276), 3 (n=89) and 8 months (n=204) after delta infection. Serum samples were collected at 3 (n=88) and 8 months (n=87) post infection from a subset of participants. Questionnaires and health care records were used to provide information about COVID vaccination and SARS-CoV-2 reinfection throughout the study period. A total of 110 participants (>16 years old) were vaccinated pre delta infection, of whom n=103 had received 1^st^ dose and n=7 the 2^nd^ dose within the defined criteria of vaccination (≥14 days prior to infection). At the 8-month follow-up, 116 participants (>15 years) were vaccinated, n=88 post delta infection, and n=28 only pre delta infection. SARS-CoV-2 reinfections, confirmed by RT-PCR or antigen tests, identified n=2 delta reinfections in Oct-Nov 2021 and n=111 omicron reinfections from 30th Dec 2021 to April 2022. The illustration was created with BioRender.

# **Supplementary figure 2: Sequence alignment in the SARS-CoV-2 receptor binding domain (RBD) regions of Wuhan, delta and omicron (BA.1 and BA.2).**

| Variant | Amino acids | aa |
| --- | --- | --- |
| Wuhan | N I T N L C P F G E V F N A T R F A S V Y A W N R K R I S N C V A D Y S V L Y N S A S F S T F K C Y G V S P T K L N D L | 390 |
| Delta | . . . . . . . . . . . . . . . . . . . . . . . . . . . . . . . . . . . . . . . . . . . . . . . . . . . . . . . . . . . . | 390 |
| BA.1 | . . . . . . . . D . . . . . . . . . . . . . . . . . . . . . . . . . . . . . . . L . P . F . . . . . . . . . . . . . . . | 390 |
| BA.2 | . . . . . . . . D . . . . . . . . . . . . . . . . . . . . . . . . . . . . . . . F . P . F A . . . . . . . . . . . . . . | 390 |
| Wuhan | C F T N V Y A D S F V I R G D E V R Q I A P G Q T G K I A D Y N Y K L P D D F T G C V I A W N S N N L D S K V G G N Y N | 450 |
| Delta | . . . . . . . . . . . . . . . . . . . . . . . . . . . . . . . . . . . . . . . . . . . . . . . . . . . . . . . . . . . . | 450 |
| BA.1 | . . . . . . . . . . . . . . . . . . . . . . . . . . N . . . . . . . . . . . . . . . . . . . . . . K . . . . . S . . . . | 450 |
| BA.2 | . . . . . . . . . . . . . . N . . S . . . . . . . . N . . . . . . . . . . . . . . . . . . . . . . K . . . . . . . . . . | 450 |
| Wuhan | Y L Y R L F R K S N L K P F E R D I S T E I Y Q A G S T P C N G V E G F N C Y F P L Q S Y G F Q P T N G V G Y Q P Y R V | 510 |
| Delta | . R . . . . . . . . . . . . . . . . . . . . . . . . K . . . . . . . . . . . . . . . . . . . . . . . . . . . . . . . . | 510 |
| BA.1 | . . . . . . . . . . . . . . . . . . . . . . . . . N K . . . . . A . . . . . . . . K . . S . R . . Y . . . H . . . . . | 510 |
| BA.2 | . . . . . . . . . . . . . . . . . . . . . . . . . N K . . . . . A . . . . . . . . R . . . . R . . Y . . . H . . . . . | 510 |
| Wuhan | V V L S F E L L H A P A T V C G P K K | 529 |
| Delta | . . . . . . . . . . . . . . . . . . . | 529 |
| BA.1 | . . . . . . . . . . . . . . . . . . . | 529 |
| BA.2 | . . . . . . . . . . . . . . . . . . . | 529 |

Amino acids (aa) that match the reference (Wuhan) are marked with dots.

Three additional mutations outside the antibody epitopes were introduced in the BA.1 and BA.2 RBD sequence to improve yield and stability (Ellis D, Brunette N, Crawford KHD, Walls AC, Pham MN, Chen C, et al. Stabilization of the SARS-CoV-2 Spike Receptor-Binding Domain Using Deep Mutational Scanning and Structure-Based Design. Front Immunol. 2021;12:710263.)

# **Supplementary figure 3. Acute and persistent symptoms in vaccinated and unvaccinated adolescents.**

#
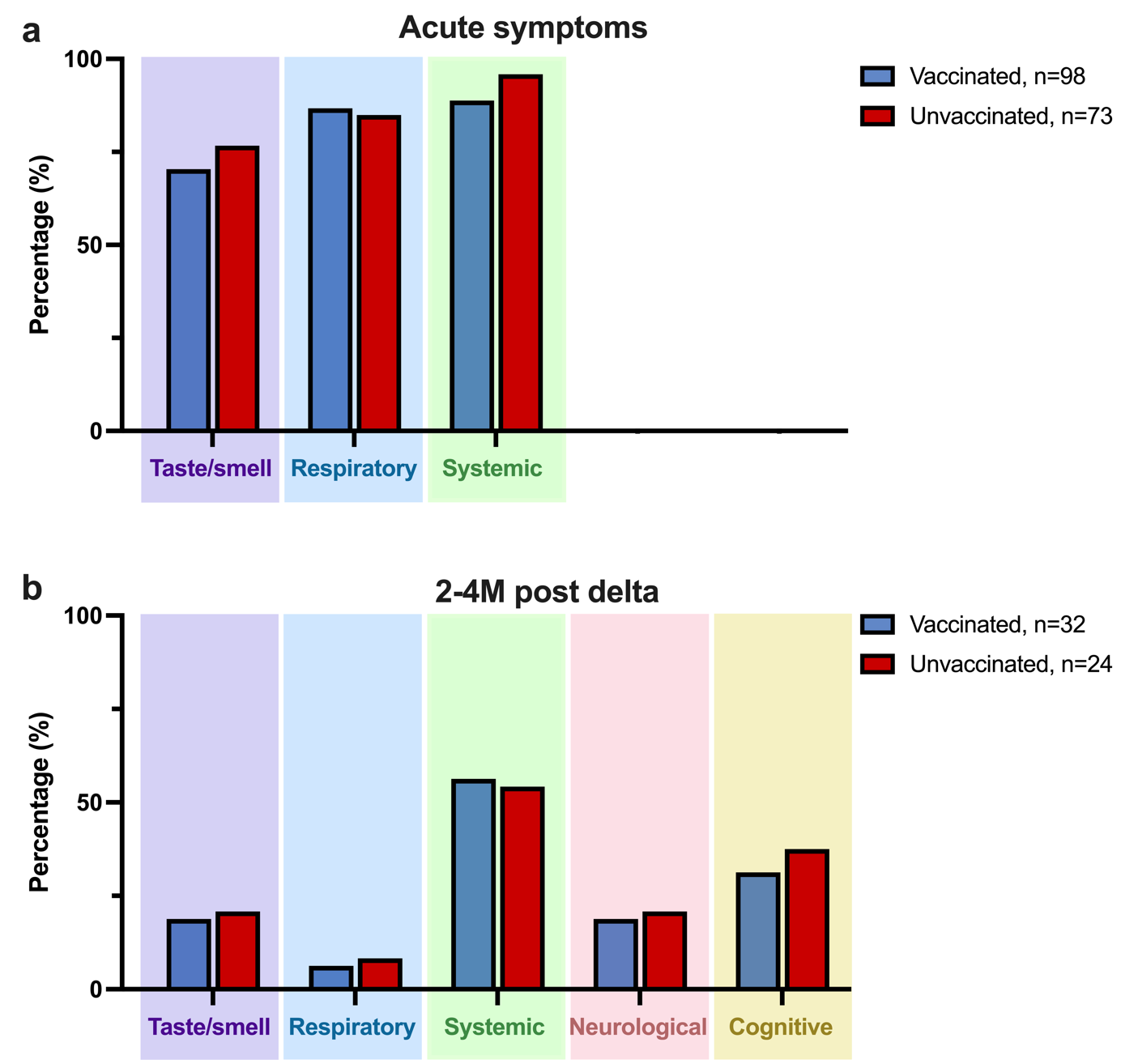


Proportion of acute a and persisting symptoms 3 months (3M) b post delta infection divided by vaccination status. Respiratory symptoms include dyspnoea, cough, congested nose/sore throat and chest pain. Systemic symptoms include fever, fatigue, headache, gastro-intestinal symptoms, muscle/joint pain and palpitations. Neurological symptoms include numbness, dizziness and sleeping problems. Cognitive symptoms include impaired memory and concentration. Vaccinated subjects are indicated in blue, and unvaccinated subjects in red. Questions about cognitive and neurological symptoms were not asked at baseline (acute symptoms).

#

# 
