# Supplementary material for: Post COVID-19 condition after delta infection and omicron reinfection in children and adolescents
Source: eBioMedicine. 2023 May 5;92:104599. doi: 10.1016/j.ebiom.2023.104599 (PMC10166589; doi:10.1016/j.ebiom.2023.104599)
Supplement: Consortia authors [file mmc2.docx]

**Bergen COVID-19 research group**

| **First names** | **Surnames** |
| --- | --- |
| Kjell | Haug |
| Helene | Sandnes |
| Kristin G-I | Mohn |
| Jan Stefan | Olofsson |
| Marianne | Sævik |
| Christopher James | Brokstad |
| Kanika | Kuwelker |
| Kristin | Heienberg |
